# Associations of body composition with physical activity, nutritional intake status, and chronotype among female university students in Japan

**DOI:** 10.1186/s40101-024-00360-9

**Published:** 2024-05-09

**Authors:** Kazushige Oshita, Yujiro Ishihara, Kohei Seike, Ryota Myotsuzono

**Affiliations:** 1 Department of Human Information Engineering, Okayama Prefectural University, Soja, Okayama 719-1197 Japan; 2 Center for Fundamental Education, Okayama University of Science, Okayama, Japan; 3 Department of Sport Science, Kyushu Kyoritsu University, Kitakyushu, Japan

**Keywords:** Skeletal muscle mass index, Body fat, Appendicular muscle mass, Morning type, Evening type

## Abstract

**Background:**

Having higher muscle mass in early adulthood is an important factor in preventing sarcopenia. However, university students undergo lifestyle changes compared to their high school years, which may lead to changes in body composition, such as an increase in body fat and a decrease in muscle mass. The study aimed to investigate the association between body composition and lifestyle behaviors, including chronotype, among Japanese female university students, due to the prevalence of underweight among young females in the country.

**Methods:**

The physical activity level (PAL), daily dietary intake status, morningness-eveningness questionnaire (MEQ) score, and body composition of 230 students were assessed in this cross-sectional study. Body composition was measured using a multifrequency bioelectrical impedance analyzer, and body mass index (BMI), body fat percentage (%BF), and skeletal muscle mass index (SMI) were determined.

**Results:**

Individuals who were evening type (ET) had a higher %BF and lower SMI than those who were non-ET, but no differences in body weight or BMI were found. Although ET individuals had lower total energy intake, protein intake, and PALs than non-ETs, the differences were small. However, multiple regression analyses showed that SMI was significantly positively associated with MEQ and PAL, and %BF was significantly negatively associated with MEQ and PAL.

**Conclusion:**

These results suggest that female university students with lateness of chronotype and low physical activity have a body composition imbalance resulting in higher body fat and lower muscle mass. Therefore, young females may need to take chronotype-specific measures (especially ET individuals) to help them maintain an appropriate body composition.

## Background

Maintaining muscle mass and strength in middle-aged individuals, minimizing the decline in older age, and increasing the peak values achieved in adolescence and young adulthood have been noted to delay or prevent future sarcopenia [1, 2]. Furthermore, although exercise interventions in frail older adults play an important role in improving muscle strength and physical function, their effect on increasing muscle mass is likely limited [3]. Therefore, increasing the peak muscle mass, particularly in early adulthood, may be important for the prevention of sarcopenia. Consequently, identifying the factors associated with muscle mass acquisition in young adults is of great importance.

Among young adults, university students undergo significant lifestyle changes that are thought to affect their weight [4] and body composition [5]. Further, individuals have a time orientation, known as the chronotype, and the sharp maximum of lateness in the chronotype (i.e., becoming an evening type (ET)) occurs around the age of 20 years [6]. Recent studies have also reported an association between chronotype and body physique in university students. For instance, a study of 384 students (150 males and 234 females) from the United States found a significant increase in body mass index (BMI) with age and lateness of chronotype and later chronotypes gaining weight faster than early chronotypes [7]. Another study with a larger sample size also concluded that university students with ET were more likely to be affected by overweight/obesity [8].

In addition to BMI, several recent studies have reported associations of chronotype with body composition and lifestyle behaviors. A study of 387 female students in Saudi Arabia found no significant main effects of chronotype on body composition, nutrient intake status, and energy expenditure [9]. However, the study participants had a higher mean body fat percentage (%BF) of 36.6% and a lower mean muscle mass (skeletal muscle index; SMI of 5.7 kg/m^2^) [9], which is the almost same as the cut-off value for the diagnosis of sarcopenia in older people [2, 10]. Therefore, this study examined a student population with extremely low muscle mass and high body fat. In contrast, a study of 327 German students (58% female) found that a later chronotype was associated with higher visceral fat mass and lower muscle mass [11]. The %BF of the participants in this study was 16.2% in males and 26.5% in females, calculated from BMI and fat mass index, which falls within the normal range. Although this study suggested that this association may be related to physical activity, the nutritional status of the participants was unknown [11]. Therefore, research is needed to determine the relationship between lifestyle behaviors, including nutritional intake status, with chronotype and body composition in the general student population.

As described above, identifying the factors associated with high muscle mass in university students is an important consideration for preventing future sarcopenia. Muscle mass in the diagnosis of sarcopenia is assessed by an index calculated from the sum of the appendicular muscle mass (AMM) [2, 10], commonly referred to as SMI. It is worth noting that individuals of Asian ethnicity may have lower fat-free mass than those of other ethnicities [12]. In particular, a high proportion of young Japanese women are underweight [13, 14] and have body composition concerns, such as normal-weight obesity [15, 16], which are associated with low fat-free mass. Therefore, studies on SMI in Japanese female university students have been reported in recent years from the perspective of future prevention of low SMI [17–20]. Previous studies showed that nutritional status such as low total energy intake (TEI) and/or protein intake (PI) has been associated with SMI in female university students [17, 20]. Low current physical activity level (PAL) was also found to be associated with low SMI, regardless of previous exercise participation [19]. Further, a previous study has indicated that university students in Japan have a shorter sleep duration compared to those in other countries [21]. This may be due to socially required early wake-up and bedtime schedules, particularly among young people who have a later chronotype, which can lead to health problems caused by the mismatch between biological and social time [22]. Therefore, it is important to investigate the factors associated with SMI among Japanese female students, considering chronotype aspects. The present study examined the relationship between lifestyle factors (PAL and dietary intake) and chronotype in relation to body composition, including the SMI in Japanese female university students.

## Methods

### Participants

**Fig. 1:**
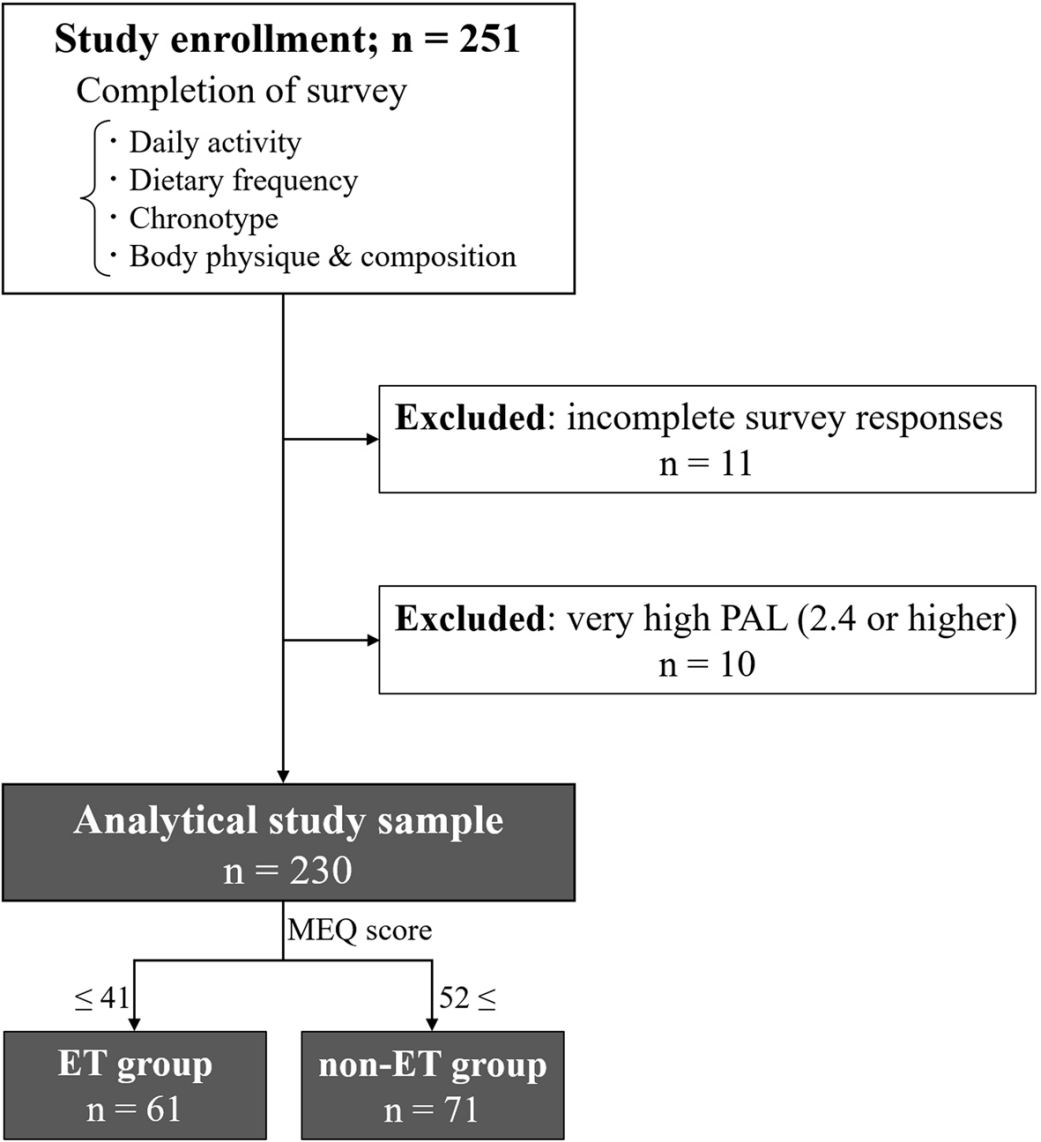
Flow diagram outlining participant selection for this study

Of the 251 total female university students, 230 participants were included in the analysis, excluding 10 with a very high PAL (2.4 or higher, PAL estimation described below) and 11 with incomplete survey responses (Fig. 1). The reasoning for exclusion based on PAL was because an extremely high PAL, such as is observed in athletes, is likely associated with high muscle mass. In addition, well-trained athletes are likely to be of the morning type (MT), although this may vary depending on the exercise [23]. The participants were recruited from one junior college and two universities in different prefectures in Japan, and the academic years were randomly chosen (aged 18–22 years). For ethical reasons, participants were informed orally and in writing in advance that the survey would be anonymous, that it would be used for the purpose of this study and not for any other purpose, and that any data not used for the study would be discarded. In the event that the results of the survey were published, participants were told that the collected data would be statistically processed and then published in such a way that individuals could not be identified, and that the survey would only be carried out if participants consented. This study was approved by the Research Ethics Committee of the Kyushu Kyoritsu University (approval number: 2022–08).

### Survey and procedures

Daily activity, dietary frequency, and chronotype surveys were conducted using a group interview method with 10–40 participants. The surveys and body composition measurements were conducted in the morning (9–12 a.m.) between late September and early December. The surveys were conducted with explanations from exercise and nutrition experts to increase the reliability of the responses.

### Estimation of PAL

The average daily activity and exercise times on weekdays in the previous month were assessed using a questionnaire-based daily activity diary. The PAL of each activity was evaluated by calculating the daily average from each classified activity using the product of the energy expenditure index, expressed as a multiple of the basal metabolic rate (BMR), and the activity time based on a study that estimated PAL according to the lifestyle of university students based on the Dietary Reference Intakes for Japanese people (DRIs-J) [19]. BMR was calculated based on height, weight, age, and sex using the National Institute of Health and Nutrition formula [24, 25]. The total energy expenditure (TEE) was calculated by multiplying the BMR by the PAL.

### Nutritional intake status and energy expenditure

The nutritional intake status and energy expenditure were assessed using a food frequency questionnaire based on food groups (FFQg) (FFQ-NEXT short version, Kenpakusha, Japan). The FFQg results were analyzed with Microsoft Excel add-in software Eiyou plus (Kenpakusha, Japan) to calculate the daily nutrient intake of each food group and the TEI, PI, fat intake (FI), and carbohydrate intake (CI). The TEI to TEE ratio (TEI/TEE) was also calculated. Although data from the UK Biobank project suggest that MTs consume more fruit and vegetables than ETs [26], the present study focused on these nutrients as factors related to body composition, as in previous studies [9, 17, 20].

### Chronotypes

The Japanese version [27] of the Horne and Ostberg morningness-eveningness questionnaire (MEQ) [28] was used to assess chronotypes. It consists of 19 questions and is scored (16–86) based on the answers. Chronotypes are scored as definitely MT (score: 70–86), moderately MT (score: 59–69), neither type (score: 42–58), moderately ET (score: 31–41), and definitely ET (score: 16–30) [28].

### Body physique and composition

Body weight and composition were measured with a body composition analyzer (MC-780, Tanita, Japan) using the multifrequency bioelectrical impedance analysis (BIA) method. The participants were asked not to urinate or defecate and to rest in a room at a comfortable temperature where they did not feel hot or cold before the measurement. The participants wiped their palms and soles with alcohol-free wet wipes to moisten and clean the electrode contact areas, stepped onto the electrode portion of the machine, and grasped the hand electrodes with both palms for measurement.

BMI was calculated by dividing body weight (kg) by the square of height (m^2^), and SMI was calculated by dividing AMM (kg) by the square of height (m^2^).

### Statistical analyses

**Fig. 2:**
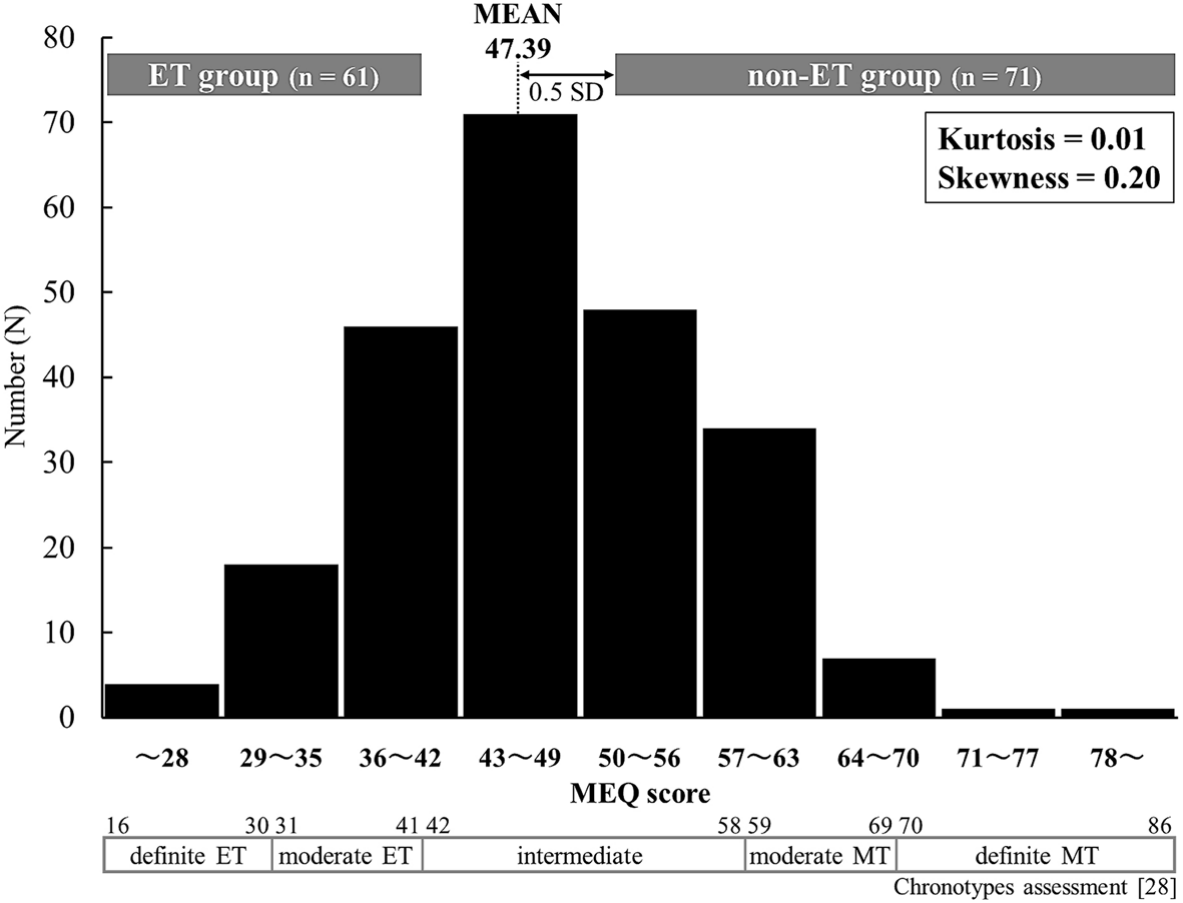
Frequency distributions of MEQ scores. (Sturges’ rule was used to calculate the number of bins in the histogram; 230 participants = 8.8≈9 bins)

The means and standard deviations (SDs) were calculated for all participants for each measure. As mentioned in the “Background” section, young individuals such as those included in the present study are expected to be predominantly ETs, and classifying them by chronotype (i.e., MT or ET) may result in fewer MTs. Consequently, following the classifications in the previous study [28] (see the “Chronotypes” section), participants with an MEQ of 41 or less were classified as the ET group. Participants with an MEQ higher than the mean of all the participants + 0.5 SD (= 47.39 + 4.68 = 52.0) were classified as the non-ET group (Figs. 1 and 2). The Mann–Whitney *U*-test was used to compare variables between the groups. Additionally, the *d*-value was calculated as the effect size using Cohen’s method.

The relationships between body composition (%BF, AMM, and SMI) and body mass, MEQ, PAL, and TEI were examined using correlation analyses. The relationships between %BF, AMM, and SMI, as dependent variables and lifestyle factors for which significant or significant trend relationships were found, were examined using forced entry multiple regression analysis.

Statistical software StatFlex (ver. 7.0.10, Artec, Osaka, Japan) was used for these statistical procedures, with a statistical significance level of *P* ≤ 0.05 and 5% < *P* ≤ 10% for a significant trend. Effect sizes were graded as *d* < 0.2 trivial effect, *d* = 0.2–0.5 small effect, *d* = 0.5–0.8 moderate effect, and 0.8 < *d* large effect [29].

## Results

Measurements for all the participants are shown in Table 1 (left), and the chronotype distribution is shown in Fig. 2. Although the distribution of chronotypes for the study participants had a kurtosis of 0.01, the skewness was 0.20, indicating that the distribution was slightly skewed toward lower MEQ scores (Fig. 2). Sixty-one participants were designated as the ET group (mean MEQ score; 36.0 ± 4.0), and 71 participants were designated as the non-ET group (58.3 ± 5.3).

**Table 1:** Mean value of each variable for all participants and ET and non-ET groups

	**All** (*n* = 230)			**ET group** (*n* = 61)			**Non-ET group** (*n* = 71)			**ET vs. non-ET**	
										***P***	**Effect size (*d*)**
**Age (years)**	**18.8**	** ± **	**1.0**	**18.7**	** ± **	**0.8**	**18.8**	** ± **	**0.8**	**0.28**	**0.06**
**Height (cm)**	**157.5**	** ± **	**5.7**	**156.9**	** ± **	**5.2**	**157.8**	** ± **	**5.4**	**0.39**	**0.08**
**Weight (kg)**	**53.1**	** ± **	**7.7**	**52.3**	** ± **	**7.5**	**53.6**	** ± **	**6.4**	**0.20**	**0.09**
**BMI (kg/m**^**2**^**)**	**21.4**	** ± **	**2.6**	**21.2**	** ± **	**2.6**	**21.5**	** ± **	**2.3**	**0.25**	**0.06**
**%BF (%)**	**28.8**	** ± **	**5.6**	**29.9**	** ± **	**4.8**	**27.3**	** ± **	**5.5**	**0.01**	**0.22**
**AMM (kg)**	**16.3**	** ± **	**2.4**	**15.8**	** ± **	**2.1**	**17.0**	** ± **	**2.5**	**< 0.01**	**0.21**
**SMI (kg/m**^**2**^**)**	**6.55**	** ± **	**0.74**	**6.41**	** ± **	**0.56**	**6.81**	** ± **	**0.86**	**< 0.01**	**0.22**
**MEQ score**	**47.4**	** ± **	**9.4**	**36.0**	** ± **	**4.0**	**58.3**	** ± **	**5.3**		
**PAL**	**1.72**	** ± **	**0.26**	**1.68**	** ± **	**0.26**	**1.76**	** ± **	**0.25**	**0.04**	**0.14**
**TEI (kcal/day)**	**1781**	** ± **	**519**	**1708**	** ± **	**582**	**1858**	** ± **	**490**	**0.09**	**0.14**
**TEI/TEE**	**0.89**	** ± **	**0.30**	**0.88**	** ± **	**0.32**	**0.90**	** ± **	**0.29**	**0.83**	**0.03**
**PI (g/day)**	**59.2**	** ± **	**20.1**	**54.9**	** ± **	**20.6**	**62.1**	** ± **	**20.5**	**0.04**	**0.16**
**FI (g/day)**	**62.9**	** ± **	**22.8**	**61.1**	** ± **	**24.9**	**66.6**	** ± **	**24.5**	**0.24**	**0.10**
**CI (g/day)**	**237.5**	** ± **	**67.4**	**227.6**	** ± **	**75.7**	**246.1**	** ± **	**58.2**	**0.11**	**0.14**

Values are expressed as means ± SDs

The measurements for each group are presented in Table 1 (right). Participant characteristics including age, height, weight, and BMI did not differ significantly between the groups. Regarding body composition, the %BF of ETs was significantly lower (27.3 ± 5.5%) than that of non-ETs (29.9 ± 4.8%). Furthermore, the AMM and SMI of non-ETs (17.0 ± 2.5 kg and 6.8 ± 0.9 kg/m^2^) were significantly higher than those of ETs (15.8 ± 2.1 kg and 6.4 ± 0.6 kg/m^2^). However, the differences were small (*d* = 0.21 and 0.22). These results indicate that ET individuals had higher body fat and lower muscle mass than those with higher MEQ scores, although no differences in body physique were found, and the measured differences were negligible.

With respect to physical activity, the PAL of non-ETs (1.76 ± 0.25) was significantly higher than that of ETs (1.68 ± 0.26), but the effect size was trivial (*d* = 0.14). This finding indicates that ET individuals are physically less active than those with higher MEQ scores, but the difference is slight.

The daily dietary intake status showed that although the TEI of non-ETs (1858 ± 490 kcal/day) tended to be significantly higher than that of ETs (1708 ± 582 kcal/day), the TEI/TEE did not differ significantly between the groups. Moreover, the FI and CI were not significantly different between the groups. Furthermore, although the PI of non-ETs (62.1 ± 20.5 g/day) was significantly higher than that of ETs (54.9 ± 20.6 g/day), the effect size was trivial (*d* = 0.16). Thus, the results indicate that although ET individuals had lower energy and protein intakes, the differences were slight.

Table 2 presents the results of the correlation analyses. %BF showed a significant positive correlation with body weight and BMI and a significant negative correlation with MEQ and PAL. AMM and SMI showed a significant positive correlation with weight, BMI, MEQ, PAL, and TEI (significant trend in relationship between SMI and TEI). The results of the multiple regression analyses are presented in Table 3. Analyses of %BF with MEQ and PAL (*R*^2^ = 0.11, *P* < 0.01) showed that %BF had a significant negative relationship with MEQ and PAL. Analysis of AMM in relation to MEQ, PAL, and TEI (*R*^2^ = 0.21,* P* < 0.01) showed that AMM had a significant positive association with MEQ and PAL. Analysis of SMI by MEQ, PAL, and TEI (*R*^2^ = 0.17, *P* < 0.01) showed that SMI was significantly positively associated with MEQ and PAL. These relationships were similar when body weight was added to the modeled variable, and %BF, AMM, and SMI were significantly positively associated with body weight, respectively.

**Table 2:** The relationships between body composition (%BF, AMM, and SMI) and body mass, MEQ, PAL, and TEI

		**Weight**	**BMI**	**MEQ**	**PAL**	**TEI**
**%BF**	***r***	**0.57**	**0.75**	**− 0.17**	**− 0.30**	**− 0.11**
	***P***	**< 0.01**	**< 0.01**	**< 0.01**	**< 0.01**	**0.11**
**AMM**	***r***	**0.68**	**0.40**	**0.21**	**0.43**	**0.13**
	***P***	**< 0.01**	**< 0.01**	**< 0.01**	**< 0.01**	**0.05**
**SMI**	***r***	**0.55**	**0.50**	**0.25**	**0.36**	**0.11**
	***P***	**< 0.01**	**< 0.01**	**< 0.01**	**< 0.01**	**0.10**

**Table 3:** Results of the multiple regression analysis

**Dependent variable: %BF**					
Modeled variable	*β*	Std. *β* (95% *CI*)	Modeled variable	*β*	Std. *β* (95% *CI*)
MEQ*	**− 0.04**	**− 0.13** (− 0.25 ~ − 0.01)	Weight**	**0.48**	**0.66** (0.57 ~ 0.75)
PAL**	**− 1.38**	**− 0.29** (− 0.41 ~ − 0.16)	MEQ**	− **0.10**	− **0.17** (− 0.26 ~ − 0.08)
			PAL**	− **8.81**	− **0.41** (− 0.50 ~ − 0.31)
***R***^**2**^** = 0.11, *F* = 14.17, *P*< 0.01**			***R***^**2**^** = 0.53, *F* = 84.60, *P* < 0.01**		
**Dependent variable: AMM**					
Modeled variable	*β*	Std. *β* (95% *CI*)	Modeled variable	*β*	Std. *β* (95% *CI*)
MEQ*	**0.04**	**0.14** (0.02 ~ 0.25)	Weight**	**0.19**	**0.61** (0.52 ~ 0.70)
PAL**	**3.78**	**0.40** (0.28 ~ 0.52)	MEQ*	**0.02**	**0.10** (0.01 ~ 0.18)
**TEI**	**< 0.01**	**0.07** (− 0.05 ~ 0.19)	PAL**	**2.77**	**0.29** (0.20 ~ 0.38)
			**TEI**	**< 0.01**	**0.06** (− 0.03 ~ 0.15)
***R***^**2**^** = 0.21, *F* = 19.74, *P* < 0.01**			***R***^**2**^** = 0.56, *F* = 73.01, *P* < 0.01**		
**Dependent variable: SMI**					
Modeled variable	*β*	Std. *β* (95% *CI*)	Modeled variable	*β*	Std. *β* (95% *CI*)
MEQ**	**0.01**	**0.19** (0.07 ~ 0.31)	Weight**	**0.05**	**0.49** (0.39 ~ 0.60)
PAL**	**0.94**	**0.33** (0.20 ~ 0.45)	MEQ**	**0.01**	**0.15** (0.05 ~ 0.26)
**TEI**	**< 0.01**	**0.05** (− 0.07 ~ 0.17)	PAL**	**0.69**	**0.24** (0.14 ~ 0.35)
			**TEI**	**< 0.01**	**0.04** (− 0.06 ~ 0.15)
***R***^**2**^** = 0.17, *F* = 15.33, *P* < 0.01**			***R***^**2**^** = 0.40, *F* = 37.34, *P* < 0.01**		

^*^P ≤ 0.05

^**^P < 0.01

## Discussion

The distribution of chronotypes was slightly skewed toward lower MEQ scores (Fig. 2). This finding suggests that many of the students in this study had lower MEQ scores (i.e., ETs), as previous research has shown that peak chronotype lateness occurs around the age of 20 [6]. The mean MEQ score for non-ETs was 58.3, which is “neither type” close to MT [28]. The mean SMI for all participants in this study was 6.55 kg/m^2^, which was slightly higher than the mean of 6.4 ± 0.5 kg/m^2^ found in a study of 426 female Japanese university students using the same body composition device [17]. Furthermore, although the correlation was weak, SMI was significantly positively correlated with the MEQ, and the SMI of ETs was significantly lower than that of non-ETs. On the other hand, the %BF results were the opposite, i.e., %BF was significantly negatively correlated with MEQ and was higher in ETs than in non-ETs. Therefore, ET individuals have an imbalance in body composition resulting in less muscle mass and more body fat but show no differences in body mass.

Differences in body composition are likely related to lifestyle behaviors. In previous studies, SMI was significantly associated with TEI in female university students [17] or students with high PAL [20]. However, in the present study, %BF and SMI did not significantly correlate with TEI, whereas AMM was significantly associated with TEI, although the correlation was weak (*r* = 0.13). The mean TEI in the previous study was low (1562 ± 489 kcal), suggesting that many participants could not consume sufficient nutrients for protein synthesis in accordance with their physical activity and that TEI was related to SMI [17]. The TEI in the present study was 1781 kcal for all participants, and the TEI was slightly lower than the TEE (TEI/TEE was approximately 0.9). Such a difference in energy intake may explain the discrepancy of results from this and previous studies on the association between TEI and SMI.

Regarding dietary intake and chronotype, recent systematic reviews have found that both MTs and ETs had similar energy intakes [30, 31]. In the present study, the TEI of ETs tended to be significantly lower than that of non-ETs, but the effect size was small, and no significant difference in TEI/TEE was found. These findings suggest that the difference in TEI between chronotypes is not large. However, the distribution of nutrients in the three meals may differ between chronotypes, even if the difference in the TEI is small. An ET was more likely than an MT to skip breakfast or consume fewer calories during breakfast while consuming more calories during dinner and snacks in the afternoon or at night [30–32]. Additionally, irregular meal patterns were also found to be associated with chronotype among Japanese university students [33]. Therefore, they may have different daily intake patterns despite having the same daily energy intake. Furthermore, the frequency of breakfast intake among Japanese female university students had shown a significant positive correlation with SMI [17]. In another study, ensuring adequate protein intake for each of the three meals as well as total daily protein intake was important for high muscle mass among Japanese university students [34]. These findings suggest that a more detailed analysis of the dietary intake is required in future studies.

According to a previous review, the relationship between physical activity and chronotype among university students is inconsistent [35]. In the present study, multiple regression analyses showed that both AMM and SMI were significantly positively associated with MEQ and PAL, and %BF was significantly negatively associated with MEQ and PAL. This suggests that lateness of chronotype and lower physical activity are associated with lower muscle mass and higher body fat. However, the PALs of both groups in the present study were within the normal range of the DRIs-J classification, and the effect size was trivial. A previous study suggested that although weekday physical activity is unlikely to show differences by chronotype, weekend physical activity (i.e., free from academic/social obligations) of ETs is lower than that of MTs [36]. Thus, although only weekday PAL was assessed in this study, the difference in PAL could have been be greater if the total weekly PAL was assessed.

A similar pattern was observed for nutrient intake. The PI was significantly lower in non-ETs in this study, which was not surprising, as the TEI was also significantly lower tend (i.e., lower total food intake). However, no significant differences were observed between the groups in FI and CI, suggesting that these intakes may have been relatively higher in ETs. In previous studies, the intake of lipids, carbohydrates, sodium, and polyunsaturated fatty acids [32] or sucrose and saturated fatty acids [37] was higher in ETs than in MTs, and a later chronotype was associated with a lower overall dietary quality index [38]. Furthermore, this difference was more pronounced on weekends [37]. Therefore, examining not only daily PAL and dietary intake but also their variation over the week would provide more evidence of the relationship between lifestyle and body composition, considering chronotype.

Previous studies have suggested that the association between BMI and chronotype may vary based on factors such as location, age, and gender [9], as well as among different racial/ethnic groups [7]. However, our study with Japanese female students found that body composition was related to chronotype and physical activity, similar to a previous study with German students (which did not investigate nutrient intakes) [11]. It is therefore possible that this relationship may be observed across racial/ethnic groups. However, it is important to note that studies have reported variations in chronotype among different racial/ethnic groups, with a higher prevalence of MT in blacks compared to whites [39]. As the chronotype of the present study population skewed toward the later side, different findings might have been observed in a population with a greater prevalence of MT. In addition, although this study was conducted during the almost autumn season in Japan (late September to early December), chronotype variation has been reported in various seasons [40]. Seasonal differences should therefore be considered in future research.

This study had several limitations. First, because PAL, nutrient intake, and chronotype were assessed using a questionnaire, future research will need to assess these factors objectively. In particular, chronotype must be assessed using clock gene analysis, as a previous study suggested that the intra-week variation in physical activity differed between clock genes and questionnaire-based assessments of chronotype [36]. Secondly, social jetlag (SJL), which refers to the difference in the midpoint of sleep between work/school days and free days, is a topic of interest in chronobiology and health research. In the Japanese population, a late chronotype has been found to be associated with an increase in SJL, and females may be at higher risk of developing adverse health symptoms because they have a higher SJL than males [22]. A previous study found that having electronic devices in the bedroom and using the Internet before going to bed are associated with SJL [41]. Although the participants in the present study attended university during the day, their nocturnal activity remains unknown. As a recent report has shown that an increase in SJL is linked to less physical activity in Japanese female students [42], future research should investigate sleep status and night-time activities as well as chronotype. Finally, as this was a cross-sectional study conducted at three universities with a limited number of participants, future research should examine changes in body composition in relation to changes in chronotypes and lifestyle behaviors. Although the sample size in this study was similar to that in recent studies on the association between chronotype and body composition in university students [7, 9, 11], further research is required to investigate this association at more universities with a larger number of participants.

## Conclusions

This study examined the relationships between physical activity, dietary intake, chronotype, and body composition in Japanese female university students. ET individuals had higher body fat and lower AMM and SMI than those with high MEQ scores, but no differences in body mass were found. Although ET individuals had lower PAL, TEI, and PI than those with high MEQ scores, the differences were small. However, multiple regression analyses showed that %BF was negatively associated with MEQ and PAL, whereas AMM and SMI were positively associated with MEQ and PAL. These results suggest that lateness of chronotype and low physical activity are associated with higher body fat and lower muscle mass in female university students. Previous reports have shown that people around the age of 20, such as university students, have peak chronotype lateness [6]. Further, university students undergo lifestyle changes compared to their high school years, which may lead to changes in body weight [4] or composition [5]. Therefore, chronotype-specific interventions (especially for ET individuals) are necessary among young females to help maintain an appropriate body composition.

## Data Availability

The datasets generated and analyzed during the current study are available from the corresponding author upon reasonable request.

## Abbreviations

SMI: Skeletal muscle mass index
AMM: Appendicular muscle mass
PAL: Physical activity level
TEI: Total energy intake
PI: Protein intake
ET: Evening type
BMR: Basal metabolic rate
DRIs-J: Dietary Reference Intakes for Japanese people
TEE: Total energy expenditure
FFQg: Food frequency questionnaire based on food groups
FI: Fat intake
CI: Carbohydrate intake
MEQ: Morningness-eveningness questionnaire
MT: Morning type
BIA: Bioelectrical impedance analysis
BMI: Body mass index
SD: Standard deviation
std β: Standard β
SJL: Social jetlag
